# *Cryptococcus* and Phagocytes: Complex Interactions that Influence Disease Outcome

**DOI:** 10.3389/fmicb.2016.00105

**Published:** 2016-02-09

**Authors:** Chrissy M. Leopold Wager, Camaron R. Hole, Karen L. Wozniak, Floyd L. Wormley

**Affiliations:** ^1^Department of Biology, The University of Texas at San AntonioSan Antonio, TX, USA; ^2^The South Texas Center for Emerging Infectious Diseases, The University of Texas at San AntonioSan Antonio, TX, USA

**Keywords:** cryptococcosis, *Cryptococcus neoformans*, *Cryptococcus gattii*, *Cryptococcus*, fungal immunity, innate immune response, medical mycology

## Abstract

*Cryptococcus neoformans* and *C. gattii* are fungal pathogens that cause life-threatening disease. These fungi commonly enter their host via inhalation into the lungs where they encounter resident phagocytes, including macrophages and dendritic cells, whose response has a pronounced impact on the outcome of disease. *Cryptococcus* has complex interactions with the resident and infiltrating innate immune cells that, ideally, result in destruction of the yeast. These phagocytic cells have pattern recognition receptors that allow recognition of specific cryptococcal cell wall and capsule components. However, *Cryptococcus* possesses several virulence factors including a polysaccharide capsule, melanin production and secretion of various enzymes that aid in evasion of the immune system or enhance its ability to thrive within the phagocyte. This review focuses on the intricate interactions between the cryptococci and innate phagocytic cells including discussion of manipulation and evasion strategies used by *Cryptococcus*, anti-cryptococcal responses by the phagocytes and approaches for targeting phagocytes for the development of novel immunotherapeutics.

## *Cryptococcus neoformans* and *C. gattii*

Cryptococcosis is a disease predominantly caused by the species *Cryptococcus neoformans* and *C. gattii* in humans and other mammals (Kwon-Chung et al., 2014). *Cryptococcus* sp. are generally encountered in the environment. *C. gattii* is commonly found in tropical and subtropical regions, however, the species is expanding its reach as evidenced by the recent outbreak in Vancouver Island, British Columbia, Canada and the Pacific Northwest of the United States (reviewed in Hole and Wormley, 2012; Kwon-Chung et al., 2014). Until recently, *Cryptococcus* sp. have been categorized into only these two species and subcategorized by serotype (A–D) and molecular type (VNI-VNIV, and VGI-VGIV; reviewed in Kwon-Chung et al., 2014). Currently, seven species of *C. gattii/C. neoformans* have been identified based on genotyping studies that have revealed significant genetic diversity within the species complex (Hagen et al., 2015). For the purposes of this review to ensure accurate reference to strains used in the discussed studies, we will refer to the organisms based on their previous *C. neoformans* (serotype A and D) and *C. gattii* (serotype B and C) nomenclature unless otherwise specified.

Interestingly, despite sharing 80–90% genomic identity (Kavanaugh et al., 2006), *C. neoformans* and *C. gattii* affect different patient populations and with different disease manifestations. The infectious propagule of both species is inhaled into the lungs and the infection is often controlled by the host’s immune system (Park and Mehrad, 2009). *C. neoformans*, however, predominantly causes disease in immunocompromised individuals, including AIDS patients and those undergoing immunosuppressive therapies, resulting in pneumonia and has a predilection to disseminate to the central nervous system (CNS) leading to life threatening meningoencephalitis (Powderly, 1993; van der Horst et al., 1997; Saag et al., 2000; Husain et al., 2001; Liu et al., 2012; Kwon-Chung et al., 2014; Sorrell et al., 2015). *C. gattii* principally causes disease in healthy individuals with no discernable underlying condition (Marr et al., 2012; Lizarazo et al., 2014). However, the idea that *C. gattii* is a primary pathogen is being questioned as anti-granulocyte-macrophage colony-stimulating factor (GM-CSF) autoantibodies have been detected in *C. gattii* infected patients but not in samples from healthy donors (Saijo et al., 2014; Kwon-Chung and Saijo, 2015). Thus, anti-GM-CSF autoantibodies could indicate an underlying immunodeficiency that predisposes patients to *C. gattii* infection. This demonstrates a need for more comprehensive screening before classifying a patient as “immunocompetent” and further examination of the *C. gattii* patient population. Another striking difference between these two species is that *C. gattii* shows little penchant for dissemination to the CNS compared to *C. neoformans* (Ngamskulrungroj et al., 2012). Comparison of two virulent strains of *C. neoformans* (strain H99) and *C. gattii* (strain R265) revealed that even though *C. gattii* can cross the blood-brain barrier (BBB), *C. neoformans* grew 10–100 times faster in naïve mouse blood and serum (Ngamskulrungroj et al., 2012). This suggests that these two species have a different specificity for their target organ that is not yet fully elucidated.

## Immune Response

The host has efficient mechanisms for combating these debilitating fungi. Initiation of a pro-inflammatory Th1-type immune response, characterized by interleukin-2 (IL-2), IL-12, IL-18, interferon-γ (IFN-γ) and tumor necrosis factor-α (TNF-α), is protective against *Cryptococcus* (Huffnagle et al., 1991; Aguirre et al., 1995; Huffnagle, 1996; Kawakami et al., 1996, 1997a; Zhang et al., 1997, 2009; Huffnagle and Lipscomb, 1998; Olszewski et al., 2010). Additionally, generation of an anti-inflammatory Th2-type immune response, characterized by IL-4, IL-5, IL-13, and the immunoregulatory cytokine IL-10, is associated with exacerbation of disease (Huffnagle et al., 1998; Arora et al., 2005, 2011; Milam et al., 2007; Muller et al., 2007; Chen et al., 2008; Jain et al., 2009; Osterholzer et al., 2009b). In addition, a Th17-type response characterized by IL-17A, IL-21, IL-22, IL-6, and TGF-β (Korn et al., 2007, 2009; Onishi and Gaffen, 2010) has been shown to contribute to anti-cryptococcal immune responses (Zhang et al., 2009; Murdock et al., 2014). However, IL-17A and signaling through the IL-17A receptor is not required for clearance in a protective model of cryptococcosis (Wozniak et al., 2011a).

Experimental studies in mice with certain *C. neoformans* strains, including the highly virulent strain H99, results in a Th2-type polarized response which is not protective (Huffnagle et al., 1998; Milam et al., 2007; Muller et al., 2007; Chen et al., 2008; Xiao et al., 2008; Osterholzer et al., 2009b). However, studies using a *C. neoformans* strain H99 that was genetically engineered to express and secrete murine IFN-γ (H99γ; Wormley et al., 2007) showed that inoculation of mice with strain H99γ results in protective Th1-type responses and IL-17 production, increased leukocyte infiltration and production of pro-inflammatory cytokines/chemokines (Wormley et al., 2007; Wozniak et al., 2009, 2011a). In addition, immunization of mice with H99γ yields 100% protection against subsequent challenge with WT *C. neoformans* H99, providing evidence that vaccination against *C. neoformans* is possible (Wormley et al., 2007; Wozniak et al., 2009; Hardison et al., 2010, 2012). Furthermore, studies examining T cell depleted mice showed that mice immunized with H99γ and challenged with WT *C. neoformans* during T cell depletion were also protected against challenge, showing that immune-compromised hosts could be protected (Wozniak et al., 2011b). The majority of studies utilize *C. neoformans* as a model organism to assess protection via Th1 and Th17-type responses, however, several studies have suggested that protection against *C. gattii* employs a different mechanism. It has been shown that infection with *C. gattii* leads to suppression of host’s immune responses, including decreased leukocyte recruitment and pro-inflammatory cytokine production (Dong and Murphy, 1995; Wright et al., 2002; Cheng et al., 2009). This could provide an indication as to how *C. gattii* is able to affect immunocompetent individuals, while *C. neoformans* predominantly causes disease in the immunocompromised populations.

It is crucial that the host immune responses will eradicate or limit the spread of fungal pathogens from the lung before they are able to disseminate. The host relies on pulmonary innate immune cells as its first line of defense against *Cryptococcus* sp. These include the phagocytic macrophages, dendritic cells (DCs) and neutrophils. When the yeast-like cell or basidiospore is encountered by the phagocyte, the phagocytic cell will engulf the invading organism forming a phagosome. Ideally, the phagosome will mature, acidify following fusion with a lysosome (forming the phagolysosome), and lead to the destruction of the fungus. *Cryptococcus* sp. have evolved a number of defense mechanisms and virulence factors that allow it to either survive in the phagosome or avoid phagocytosis altogether (reviewed in Kronstad et al., 2011; Kwon-Chung et al., 2014; Alspaugh, 2015). The capsule of *Cryptococcus* is a complex and elaborate structure composed of glucuronoxylomannan (GXM), galactoxylomannan (GalXM) and, to a lesser degree, mannoproteins and is anchored to the cell wall, which is composed of chitin, chitosan (acetylated chitin), glucans and glycoprotein (Doering, 2009; O’Meara and Alspaugh, 2012). The capsule is dynamic and dramatically increases in thickness during infection or when cultured in host inducing conditions (RPMI, 37°C). Additionally, the capsule functions to mask cryptococcal pathogen associated molecular patterns (PAMPs) that can be recognized by pattern recognition receptors (PRRs) on host cells, allowing *Cryptococcus* to evade the immune system by preventing phagocytosis and can aid in providing protection against reactive oxygen species and antimicrobial peptides (Zaragoza et al., 2008). Opsonization of *Cryptococcus* with anti-capsular antibodies is required for phagocytosis by immune cells due to the anti-phagocytic properties of the capsule (reviewed in Kozel, 1993). The ability to form “titan cells” is another anti-phagocytic mechanism utilized by *Cryptococcus*. In the pulmonary environment, *C. neoformans* cells can attain a large cell size varying from 50 to 100 μm in diameter with varying degrees of capsule thickness making them resistant to phagocytosis (Okagaki et al., 2010; Zaragoza et al., 2010; Okagaki and Nielsen, 2012). Melanin production by laccase is another virulence factor of *C. neoformans* and *C. gattii* which serves to protect the organism from oxidative stresses within the phagolysosome by breaking down host substrates into reactive intermediates that can harm the host (Zhu and Williamson, 2004; Panepinto and Williamson, 2006). One example of this is the iron oxidase activity of laccase in the phagolysosome that reduces potentially toxic Fenton reactants, thus protecting *Cryptococcus* from the antifungal activity of alveolar macrophages (Liu et al., 1999). Other virulence factors include the ability to grow and proliferate at mammalian body temperature (37°C) and extracellular enzymes including laccases, urease, and phospholipases (reviewed in Kronstad et al., 2011; Almeida et al., 2015). In this review, we discuss interactions between *Cryptococcus* sp. and innate phagocytic cells and how these interactions determine the outcome of disease.

## Macrophages

### Macrophage Activation

**FIGURE 1:**
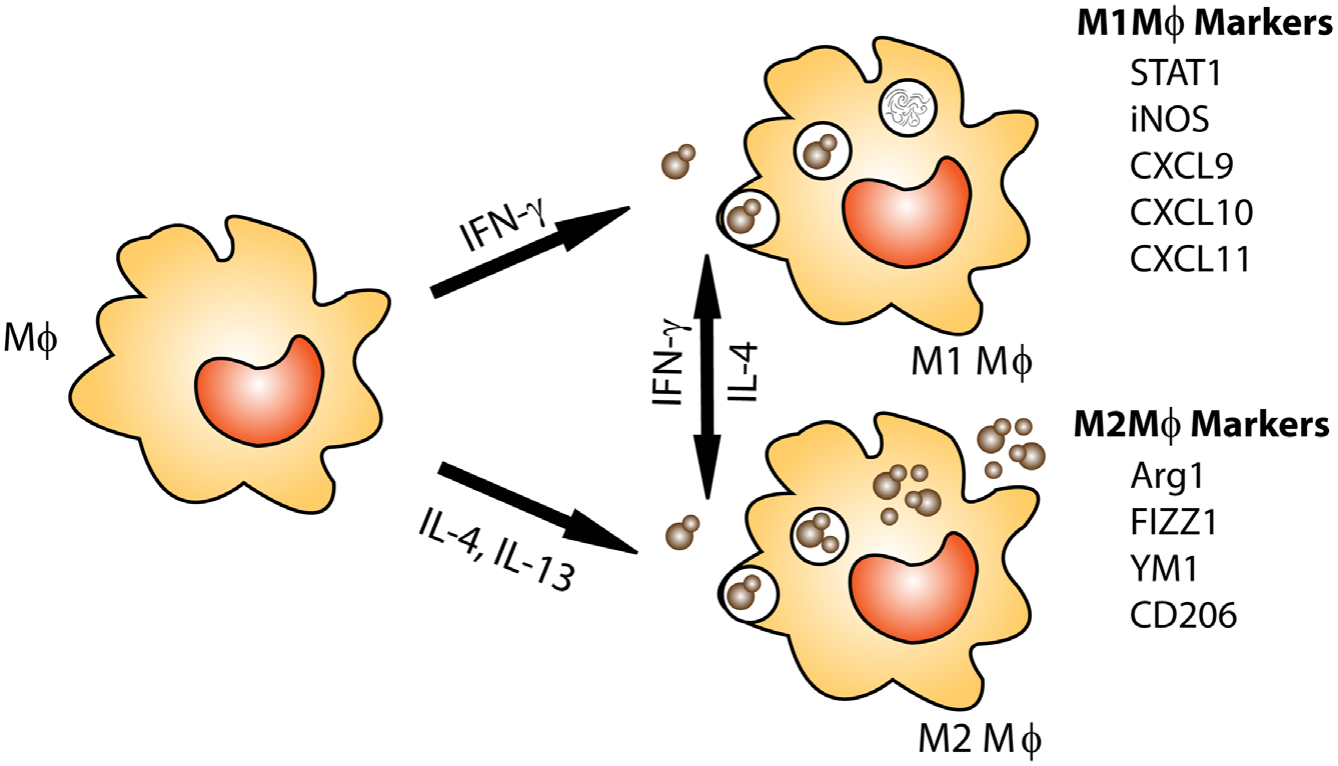
**Macrophage activation: protection against *Cryptococcus* is associated with M1 or classically activated macrophages.** M1 macrophages are induced by exposure to the Th1 cytokine IFN-γ. M1 macrophages are efficient killers as they have increased nitric oxide (NO) and ROS production. M2 or alternatively activated macrophages are associated non-protective immune responses to *Cryptococcus*. M2 macrophages are not efficient killers and allow *Cryptococcus* to grow and proliferate within the macrophage and may lead to dissemination. In addition, macrophage phenotype is plastic, allowing for macrophages to switch between M1 and M2 phenotypes based on cytokines present in their environment.

Resident alveolar macrophages which live within the lung alveolar airspaces are critical to host defense as the first line of defense against pulmonary pathogens (Fels and Cohn, 1986). Alveolar and infiltrating macrophages phagocytose and kill invading pathogens and are capable of presenting antigen to activated T cells to stimulate an adaptive immune response in immunocompetent individuals. Interestingly, macrophages have a dynamic plasticity which allows them to respond to changes in their cytokine microenvironment and alter their activation phenotype (Porcheray et al., 2005; Stout et al., 2005; Gratchev et al., 2006; Sica and Mantovani, 2012; Davis et al., 2013; Mantovani et al., 2013; Leopold Wager and Wormley, 2014). Macrophage activation phenotype is broadly classified as classical (M1) or alternative (M2) based on cytokine production, extracellular receptor expression and secreted by-products (Stout and Suttles, 2004; Mosser and Edwards, 2008; Sica and Mantovani, 2012). Immune cells, including natural killer (NK) cells, CD8^+^ T cells, and CD4^+^ T-helper 1 (Th1)-type cells (Mosser and Edwards, 2008) respond to the pathogen by secreting inflammatory cytokines, including IFN-γ which signals macrophages to polarize toward an M1 phenotype (Mantovani et al., 2004; Mosser and Edwards, 2008; Martinez et al., 2009; Hussell and Bell, 2014). The markers commonly used for identification of M1 macrophages include inducible nitric oxide synthase (iNOS), chemokine (C-X-C motif) ligand 9 (CXCL9), CXCL10, CXCL11, IL-12, and suppressor of cytokine signaling 3 (SOCS3; reviewed in Mosser and Edwards, 2008; Murray and Wynn, 2011; Leopold Wager and Wormley, 2014). M1 macrophages mediate host defense against microbial pathogens via the generation of reactive oxygen and nitrogen species (ROS and RNS, respectively; Ding et al., 1988). The enzyme iNOS acts on the substrate L-arginine to produce nitric oxide (NO), which has anti-cryptococcal properties (**Figure 1**; Alspaugh and Granger, 1991; Arora et al., 2011; Hardison et al., 2012; Davis et al., 2013; Leopold Wager et al., 2014, 2015). Conversely, the enzyme arginase-1 (Arg-1) is a hallmark marker of M2 macrophage activation and competes with iNOS for this substrate producing L-ornithine and urea (Bogdan et al., 2000). The Arg1 to iNOS ratio is often considered an indicator of macrophage polarization phenotype as conditions leading to the induction of M2 macrophages inhibit M1 macrophage activation (Takeda et al., 1996; Van Dyken and Locksley, 2013).

The induction of M2 macrophage activation is mediated by cytokines including IL-4 and IL-13 (Mosser and Edwards, 2008; Mantovani et al., 2013; Van Dyken and Locksley, 2013). It was recently shown that cryptococcal Ssa1, a heat shock protein 70 homolog, is secreted by *C. neoformans* and promotes early fungal growth and a shift to M2 macrophage activation by stimulating early production of IL-4 and IL-13 (Eastman et al., 2015). M2 macrophages contribute to the suppression and regulation of inflammatory responses and play a pivotal role in wound-healing, but are not antimicrobial against *C. neoformans* (Arora et al., 2005, 2011; Mosser and Edwards, 2008; Martinez et al., 2009; Hardison et al., 2010, 2012; Murray and Wynn, 2011; Davis et al., 2013; Van Dyken and Locksley, 2013; Leopold Wager and Wormley, 2014; Leopold Wager et al., 2014, 2015). *C. neoformans* cells can survive in M2 macrophages by using them as a protective niche to evade recognition and killing by the host (reviewed in Johnston and May, 2013). Immune cells including Th2-type CD4^+^ T cells, basophils, eosinophils, mast cells and group 2 innate lymphoid cells (ILC2s) are sources of IL-4 and/or IL-13 (Martinez et al., 2009; Murray and Wynn, 2011; Walker and McKenzie, 2013; Walker et al., 2013). Recently, it was shown that chitin, an integral part of the cryptococcal cell wall, indirectly stimulates the production of IL-5 and IL-13 and can trigger IL-4 production, resulting in M2 macrophage activation (Reese et al., 2007; Van Dyken et al., 2014). IL-4 can stimulate increased expression of mannose receptor (MR or CD206), the first described M2 macrophage activation-associated marker (Stein et al., 1992). The commonly associated markers of M2 macrophage activation also include arginase1 (Arg1), found in inflammatory zone 1 (FIZZ1, also known as resistin-like α or Relm-α), and chitinase and chitinase-like molecules such as YM1 (Chi3l3) and YM2 (Mosser and Edwards, 2008; Murray and Wynn, 2011).

Interestingly, macrophages, which are polarized toward an M1 phenotype in the presence of IFN-γ, can re-polarize to an M2 phenotype when stimulated with IL-4. Similarly, M2 polarized macrophages can re-polarize toward a functional M1 phenotype in the presence of IFN-γ and are able to maintain functional anti-cryptococcal activity (Stout et al., 2005; Gratchev et al., 2006; Davis et al., 2013), signifying the critical importance of the local cytokine milieu in driving macrophage polarization. IFN-γ production by Th1-type T cells and NK cells can stimulate M1 macrophage activation in a signal transducer and activator of transcription (STAT) 1-dependent manner (Hu et al., 2002). Studies utilizing the IFN-γ producing *C. neoformans* strain have illustrated that STAT1 signaling in macrophages is required for M1 macrophage activation and protection (Leopold Wager et al., 2014; Leopold Wager et al., 2015). More in-depth discussion of the macrophage polarization and *C. neoformans* infections has been recently published elsewhere (Leopold Wager and Wormley, 2014). Conversely, a clinical study recently showed that despite elevated IFN-γ levels and pro-inflammatory factors in the brains of two non-HIV cryptococcal meningitis patients, poor phagocytosis and macrophages polarized to the non-protective M2 phenotype was observed (Panackal et al., 2015). This establishes that macrophage polarization in response to *C. neoformans* in humans is quite complex and requires further study.

*Cryptococcus neoformans* is sensitive to the NO produced by M1 macrophages (Alspaugh and Granger, 1991; Aguirre and Gibson, 2000; Rivera et al., 2002; Muller et al., 2007; Osterholzer et al., 2009b; Stenzel et al., 2009; Zhang et al., 2009; Hardison et al., 2010). *C. neoformans* is thought to down-regulate macrophage iNOS RNA, thus mediating NO suppression (Kawakami et al., 1997b; Chinen et al., 1999). Inhibition of iNOS results in M2 macrophage activation, loss of anti-cryptococcal activity and progression of disease (Naslund et al., 1995; Arora et al., 2005, 2011; Xiao et al., 2008; Hardison et al., 2010; Leopold Wager et al., 2015). In addition, macrophages from iNOS deficient mice or from WT mice cultured with iNOS inhibitors are unable to control the intracellular proliferation of *C. neoformans*, even in the presence of intact ROS production (Leopold Wager et al., 2015). This reveals that NO, and not ROS, is the mechanism used by M1 macrophages to control *C. neoformans* in mice. However, how well this finding translates to human macrophages is a point of contention as the human iNOS gene is generally epigenetically silenced by CpG methylation, histone modifications and chromatin compaction (Gross et al., 2014). Interestingly, other studies have shown granulomas from pulmonary tissues in humans and non-human primates contain iNOS^+^ macrophages (Facchetti et al., 1999; Mattila et al., 2013), suggesting that human macrophages are capable of NO production that could control cryptococcal growth. Further studies that would characterize the human macrophage response to *Cryptococcus* are required to comprehensively address this question.

### Survival and Replication in Macrophages

Following phagocytosis, cryptococci have been shown to proliferate in the macrophage phagosome (Tucker and Casadevall, 2002; Voelz et al., 2009), suggesting that these phagocytes are a preferred niche of the yeast where it can hide from recognition by the host’s immune system (Johnston and May, 2013). A clinical study recently examined 65 clinical isolates of *C. neoformans* and demonstrated that strains with high rates of phagocytosis by macrophages with low intracellular proliferation *in vitro* coincided with higher CSF fungal burdens and, paradoxically, long-term survival of HIV patients (Sabiiti et al., 2014). The strains with high uptake rates were hypocapsular, had enhanced laccase activity, and were more resistant to patient antifungal treatment (Sabiiti et al., 2014). This study demonstrates that cryptococcal-phagocyte interactions are a major contribution to human clinical presentation and outcome.

In the phagosome, cryptococci are exposed to low pH, ROS and NO. Studies have shown that *C. neoformans* does not actively avoid acidification of the phagolysosome in macrophages, but actually proliferate better in acidic rather than alkaline conditions (Diamond and Bennett, 1973; Levitz et al., 1999). Interestingly, live, but not heat killed, *C. neoformans* can induce premature removal of phagosome markers Rab5 and Rab11 (early phagosome markers; Smith et al., 2015). In contrast to previous studies, Smith et al. (2015) have recently shown that *C. neoformans* can hinder significant acidification of the phagosome, calcium flux and protease activity, rendering the phagosome permissive to cryptococcal proliferation in both the J774.1 macrophage-like cell line and human monocyte derived macrophages. This study also demonstrated that several virulence attenuated mutants are able to prevent phagosome maturation (Smith et al., 2015), suggesting that an unknown mechanism regulates this process. Studies have shown that incubation of murine macrophages with *C. neoformans* can result in host damage via activation of stress pathway including HIF-1α, receptor-interacting protein 1, apoptosis-inducing factor and mitochondrial depolarization (Coelho et al., 2015). A recent study by Davis et al. (2015) showed that live *C. neoformans* can cause lysosomal damage in bone marrow derived macrophages (BMM) which increases over time (Davis et al., 2015). Stimulation of BMMs with IFN-γ negated the lysosomal damage and increased killing of the yeast (Davis et al., 2015), suggesting that the induction of lysosomal damage is a potential survival strategy of *C. neoformans* that is counteracted when macrophages are activated to the M1 phenotype.

Phospholipase B, a known virulence factor of *Cryptococcus*, is a phospholipid modifying enzyme found at the cell surface (Djordjevic et al., 2005). This enzyme is active under acidic conditions at 37°C, as found in macrophage lysosomes. Deletion of *PLB1* in *C. neoformans* results in attenuated virulence, increased capsular diameter and size in the phagosome and in the lung (Evans et al., 2015). *PLB1* is also required for proliferation and survival in macrophages, as well as for dissemination to the CNS (Evans et al., 2015). Another cryptococcal protein, Fbp1, is critical for survival and proliferation in the macrophage phagosome and for dissemination from the lungs (Liu and Xue, 2014). Fbp1, along with its substrate inositol phosphosphingolipid-phospholipase C, is required for resistance to NO and dissemination (Liu and Xue, 2014).

*Cryptococcus neoformans* is capable of surviving even in a phagosome with ROS, in part due to absorption of ROS by the capsule (Zaragoza et al., 2008). In addition, the plasma membrane high-affinity Cch1-Mid1 calcium channel (CMC) of *C. neoformans* promotes cryptococcal survival during exposure to oxidative stress (Vu et al., 2015). Cch1 mutants have decreased survival in J774.1A macrophages, however, *C. neoformans* deficient in both Cch1 and Mid1 maintain resistance to the ROS, suggesting a compensatory mechanism in the yeast (Vu et al., 2015). *C. gattii*, however, has been shown to use a novel mechanism in response to ROS. The *C. gattii* Vancouver Island outbreak strain R265 induces to tubularization of its mitochondria, which can facilitate the growth of nearby “normal” *C. gattii* cells (Voelz et al., 2014). This enables the cryptococci to establish an intracellular niche in the macrophage, increasing pathogenesis. There was no observed correlation in *C. neoformans* between mitochondria tubularization and intracellular proliferation rate (Voelz et al., 2014), suggesting a unique mechanism of *C. gattii*.

### Trafficking Through the Blood Brain Barrier

Remarkably, *C. neoformans* is capable of non-lytic exocytosis, where the yeast cells burst out of the macrophage without lysing the phagocyte, again, preventing a response from the host (Alvarez and Casadevall, 2006; Johnston and May, 2010; Nicola et al., 2011). The ability of *Cryptococcus* to proliferate inside macrophages and then escape is the basis for one hypothesis as to how *C. neoformans* traffics across the BBB to cause meningoencephalitis in immunocompromised hosts. It has been posited that macrophages act as a “Trojan horse” carrying the yeast-like cells into the CNS while hidden from the immune system (Liu et al., 2012). Histological sections of *C. neoformans* infected brain tissue show cryptococci within macrophage-like cells either within or outside capillaries (Chretien et al., 2002). A second hypothesis is paracellular crossing of the BBB due to loss of integrity in the tight junctions or injury to the brain endothelium. These events compromise the BBB, leaving openings for the yeast cells to cross into the CNS (Ibrahim et al., 1995; Chen et al., 2003; Olszewski et al., 2004; Charlier et al., 2005). Third, the transcellular pathway is a hypothesis in which cryptococcal cells transmigrate via adherence to and internalization by the brain endothelium from the luminal surface (blood side) to the abluminal side (brain side) of the BBB (Chang et al., 2004; Liu et al., 2012; Sabiiti and May, 2012). The metalloprotease Mpr1 of *C. neoformans* is an extracellular protein that is required for migration across the BBB as it promotes adherence of the yeast cells to the brain endothelial cells (Vu et al., 2014). *C. neoformans* deficient in Mpr1 is able to survive in J774.1 macrophages (Vu et al., 2014), providing additional evidence for the transcellular pathway hypothesis.

Murine studies suggest that *C. gattii* does not traffic to the brain as readily as *C. neoformans* (Ngamskulrungroj et al., 2012), suggesting that dissemination to the CNS is not a primary target of *C. gattii*. *C. neoformans* is phagocytosed more efficiently by THP-1 macrophage-like human cells regardless of prior stimulation of the macrophage-like cells with IFN-γ (Sorrell et al., 2015). Remarkably, more *C. neoformans*-loaded macrophages traveled across the human brain endothelial cell line monolayer (a model of the BBB) than *C. gattii*-loaded macrophages (Sorrell et al., 2015). *C. neoformans* also had a higher rate of expulsion from the THP-1 macrophages (Sorrell et al., 2015), further validating the Trojan horse hypothesis. All hypotheses concerning trafficking of *C. neoformans* to the CNS are supported by strong evidence as discussed above. A likely explanation is that all mechanisms of trafficking are utilized by *C. neoformans* to gain access to this privileged site.

## Dendritic Cells

Dendritic cells are innate cells that act as sentinels of the immune system. These cells are phagocytes, but also have the ability to present antigen to naïve T cells in order to direct the adaptive immune response. Initial *in vitro* studies of DCs with *C. neoformans* showed that DCs are involved in detection, binding, phagocytosis, processing, antigen presentation, T cell activation, and killing of the organism (Bauman et al., 2000, 2003; Wozniak et al., 2006; Wozniak and Levitz, 2008). Early studies showed that Langerhans cells and myeloid DCs are necessary for the induction of protective immune responses against *C. neoformans* (Bauman et al., 2000).

Uptake of *C. neoformans* by DCs requires opsonization with either anti-capsular antibody or complement, due to the anti-phagocytic polysaccharide capsule surrounding the cryptococcal organisms (Kelly et al., 2005). In contrast to encapsulated strains, phagocytosis of acapsular mutant *C. neoformans* strains by DCs requires MR and FcγR II (Syme et al., 2002). Toll-like receptor 2 (TLR2) and TLR4 are not important in uptake of *C. neoformans* or activation of DCs by the fungus (Nakamura et al., 2006). Following phagocytosis by DCs, cryptococci translocate to the endosomal compartment followed by the lysosomal compartment, where they are killed by oxidative and non-oxidative mechanisms (Kelly et al., 2005; Artavanis-Tsakonas et al., 2006; Wozniak and Levitz, 2008; Hole et al., 2012). Studies examining DC lysosomal extract *in vitro* showed direct anti-cryptococcal activity (Wozniak and Levitz, 2008; Hole et al., 2012). Purified lysosomal enzymes, specifically cathepsin B, inhibit cryptococcal growth by cell wall damage followed by osmotic lysis of the cryptococcal cells (Hole et al., 2012).

During a cryptococcal infection in a mouse model, cryptococci are rapidly internalized by pulmonary DCs (Wozniak et al., 2006). These DCs increase surface expression of costimulatory molecules CD80 and CD86 as well as MHC II by D7 post-infection. DCs isolated from infected lungs present cryptococcal mannoprotein (MP) to MP-specific T cells and induced T cell activation and proliferation *ex vivo* (Wozniak et al., 2006). Furthermore, DC phagocytosis of cryptococcal MP in the presence of the appropriate adjuvant, such as the Th1-type inducing adjuvant CpG, induces production of protective Th1-type cytokines (Mansour et al., 2002; Dan et al., 2008a). Upon uptake of cryptococcal MP, DCs express markers of activation and maturation including MHC I and MHC II as well as CD40, CD80, and CD86 (Pietrella et al., 2005; Dan et al., 2008a,b). MP induces human DCs to secrete IL-12 and TNF-α, which are associated with the protective Th1-type immune response, and also leads to Iκβα phosphorylation (Pietrella et al., 2005). MP-loaded DCs are efficient stimulators of T cells resulting in CD4 and CD8 proliferation (Pietrella et al., 2005). Additional studies revealed that the interaction of *C. neoformans* with DCs, but not macrophages, induced the production of IL-12 and IL-23, two cytokines associated with protection against cryptococcosis (Kleinschek et al., 2010). Though macrophages are capable of presenting antigen to activated T cells, it is the DCs that are the most efficient antigen presenting cell type to present *C. neoformans* mitogen, and only a small number of DCs are needed for antigen presentation to T cells (Syme et al., 2002).

Dendritic cells and alveolar macrophages are required for protection against cryptococcal infection and are needed early in host defense (Osterholzer et al., 2009a). Depletion of DCs abrogated the T cell response in mice (Mansour et al., 2006). Depletion of CD11c^+^ cells using a CD11cDTR mouse during cryptococcal infection lead to mortality within 6 days post-infection. Death was associated with neutrophilic bronchopneumonia and alveolar damage (Osterholzer et al., 2009a). The recruitment of monocyte-derived DCs into the lung appears to be dependent on the chemokine receptor CCR2. CCR2 KO mice infected with *C. neoformans* show impaired DC recruitment and the mice developed features of a Th2-type response including persistent infection, bronchovascular collagen deposition, and increased IL-4 production (Osterholzer et al., 2008). CCR2-dependent recruitment of DCs into *C. neoformans* infected lungs was due to increased recruitment of Ly-6C^high^ monocytes that differentiate into CD11b^+^ DCs in the lungs (Osterholzer et al., 2009a). These data suggest that CCR2 is required for the recruitment of DCs to the lungs and initiation of protective immune responses during a cryptococcal infection. (Osterholzer et al., 2008).

Cryptococcal capsule prevents phagocytosis of the organism (in the absence of opsonization by complement or antibody). In addition to its role in preventing phagocytosis by DCs and other phagocytes, the main capsular polysaccharide, GXM, has profound suppressive effects on immune responses (Yauch et al., 2006; Zaragoza et al., 2009). Cryptococcal capsule interferes with both DC activation and maturation (Vecchiarelli et al., 2003; Lupo et al., 2008; Grijpstra et al., 2009). In contrast, acapsular strains phagocytosed by DCs induce surface expression of MHC II and other costimulatory molecules, whereas the encapsulated strains do not induce activation unless opsonized by an anti-GXM antibody which is recognized by CD32 and CD16 (Vecchiarelli et al., 2003). Acapsular mutant strain cap56Δ induces human DC activation as seen by increased CD80 and CD86 surface expression (Grijpstra et al., 2009). In addition to affecting DC maturation, capsular material can influence DC gene expression of several cytokines and chemokines associated with protective responses as well as genes associated with antigen presentation (Lupo et al., 2008). Encapsulated strains induce a down-regulation of cytokine genes and inhibited the induction of the genes for cytokine and chemokine production as well as antigen presentation (Lupo et al., 2008).

Although the Th1-type immune response is typically associated with protective anti-cryptococcal responses, a Th2-type response may not always be detrimental under certain conditions. Experimental pulmonary infection of IL-4Rα KO mice with a *C. neoformans* serotype D strain resulted in higher fungal burden compared to WT mice (Grahnert et al., 2014). Additionally, IL-4Rα KO mice exhibited a defect in DC and macrophage recruitment due to a reduction in CCL2 and CCL20 chemokines. There was also a reduction in IFN-γ and NO production in the IL-4Rα KO mice (Grahnert et al., 2014). *In vitro* culture of DCs in the presence of *C. neoformans* and IL-4 resulted in increased IL-12 and reduced IL-10 production by the DCs (Grahnert et al., 2014). These data in conjunction with the increased fungal burden in the IL-4Rα KO mice suggest a beneficial role of IL-4Rα signaling early during infection, whereas IL-4Rα signaling during the late phase of infection is detrimental (Grahnert et al., 2014).

Infection with virulent wild-type *C. neoformans* strains naturally induce a strong Th2-type immune response. Urease produced by *C. neoformans* directed a strong Th2-type immune response and lead to a significant increase in the amount of immature DCs that accumulated in the lung-associated lymph nodes (Osterholzer et al., 2009b). Using a tetramer for chitin deacetylase 2 (CDA2), Wiesner et al. (2015) were able to isolate *Cryptococcus*-specific Th2 CD4^+^ T cells and found that chitin recognition via chitotriosidase leads to the induction of Th2 cells by DCs. Using multiple DC KO and DC depleted mouse models, Wiesner et al. (2015) also demonstrated that lung-resident CD11b^+^ IRF4-dependent conventional DCs are responsible for the induction of Th2-type T cells. This indicates that DCs can have a protective or a non-protective role in the immune response to *C. neoformans*. The ability of DCs to react protectively or non-protectively suggests that these cells are heavily influenced by their environment, a fact that could be exploited for development of novel immunotherapies to combat cryptococcosis.

**FIGURE 2:**
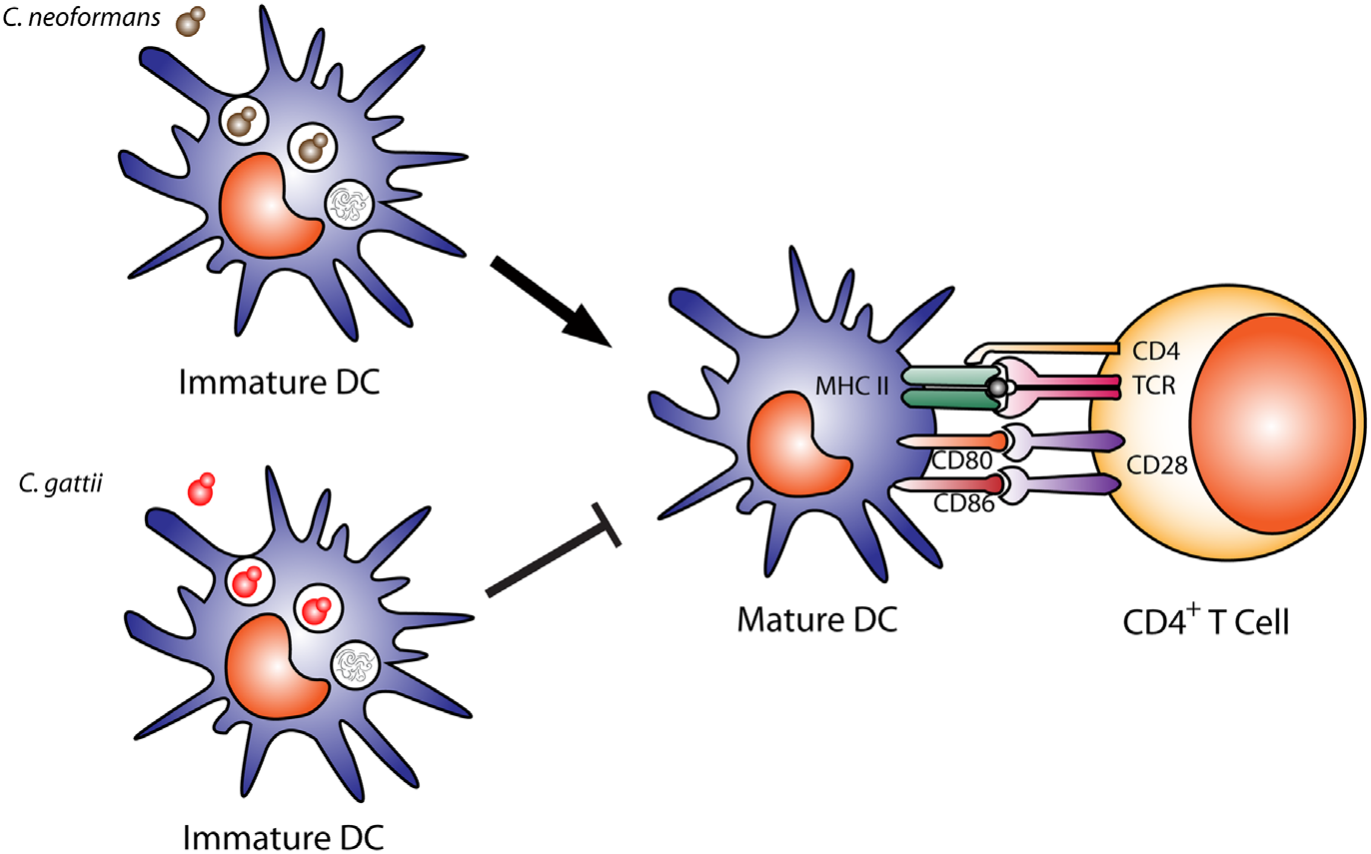
**Dendritic cell cryptococcal killing and antigen presentation.** Immature dendritic cells efficiently phagocytose and kill opsonized *Cryptococcus neoformans* cells, leading to DC maturation, shown by upregulation of costimulatory molecules (MHC II, CD80, CD86) and antigen presentation to CD4^+^ T cells. In contrast, *C. gattii* can also be phagocytosed and killed by DCs, but leads to a downregulation of DC maturation and expression of costimulatory molecules MHC II, CD80, CD86 and reduces antigen presentation to T cells.

The interaction of DCs with *C. gattii* can differ dramatically from *C. neoformans*. Human monocyte-derived DCs can kill *C. gattii*, but this does not induce DC maturation (**Figure 2**; Huston et al., 2013). Even after uptake and processing of *C. gattii* by human DCs, they do not increase expression of MHC class II, CD86, CD83, CD80, and CCR7, which results in defects in T cell responses (Huston et al., 2013). In addition, DCs that kill *C. gattii* do not trigger a release of TNF-α, which is important for cryptococcal clearance (Huston et al., 2013). In mice infected with *C. gattii*, the DCs express much lower levels of surface MHC II and IL-12 or IL-23 transcripts and fail to induce effective Th1 and Th17 differentiation *in vitro* compared to mice infected with *C. neoformans* (Angkasekwinai et al., 2014). In a DC vaccine model for *C. gattii*, uptake of an acapsular mutant by DCs induced expression of costimulatory molecules and inflammatory cytokines (Ueno et al., 2015). Mice immunized intravenously with BMDCs pulsed with this acapsular mutant and challenged with *C. gattii* R265 showed significantly less pathology, reduced fungal burden, significantly increased survival compared to controls. Immunized mice had significantly increased lung and spleen lymphocytes producing IL-17A, IFN-γ, and TNF-α compared to non-immunized mice, and protection was significantly reduced IFN-γ KO mice (Ueno et al., 2015). Altogether, prevention of DC maturation by *C. gattii* could explain how this yeast causes disease in seemingly immunocompetent individuals.

## Neutrophils

Neutrophils are phagocytes that are recruited to the lungs during cryptococcal infection and can kill *C. neoformans*. However, the importance of neutrophils in protective responses against cryptococcal infection is unclear. Human neutrophils, polymorphonuclear leukocytes (PMNs), have been shown to kill *C. neoformans in vitro* by both oxidative and non-oxidative mechanisms (Mambula et al., 2000). Treatment with inhibitors of the respiratory burst only partially reduced the anti-cryptococcal activity of human PMNs (Mambula et al., 2000). The non-oxidative anti-cryptococcal activity of human PMNs was shown to be mediated by calprotectin and defensins (Mambula et al., 2000). Mice lacking myeloperoxidase (MPO), an enzyme associated with neutrophil antimicrobial activity, succumb to the infection faster than WT mice upon infection with *C. neoformans* either intranasally or intravenously (Aratani et al., 2006). *In vitro*, neutrophils migrate toward cryptococcal cells using the complement C5a-C5aR pathway, and the interaction leads to phagocytosis and killing of *C. neoformans* and enhances activation of Erk and p38 mitogen activated protein kinases (MAPK) in neutrophils (Sun et al., 2016). Interestingly, inhibition of p38 MAPK pathway significantly decreased neutrophil migration and cryptococcal killing (Sun et al., 2016). Further studies showed that neutrophils can migrate into the brain microvasculature, phagocytose the cryptococcal cells before they are able to migrate into the brain parenchyma, and re-enter the circulation, effectively removing the cryptococci from the brain (Zhang et al., 2015b).

While neutrophils can kill *C. neoformans*, the pathogen can also modulate the neutrophil response. Cryptococcal capsular and cell wall components can inhibit neutrophil migration (Coenjaerts et al., 2001; Ellerbroek et al., 2004) and can inhibit the production of neutrophil extracellular traps (NETs; Rocha et al., 2015). In addition, melanized *C. neoformans* cells are able to abrogate the killing activity of neutrophils by interfering with sphingomyelin synthase (SMS) activity, which is required for cryptococcal killing by neutrophils (Qureshi et al., 2010, 2011). Cryptococcal cells can prevent neutrophil migration by secreting capsular components, which activates microglia to produce IL-8 (a neutrophil chemoattractant) and also reduces the expression of L-selectin (CD62L) on the neutrophil surface in humans with disseminated cryptococcosis, reducing neutrophil migration. Neutrophil endothelial rolling and production of surface expression of TNF-α receptor are also impaired due to cryptococcal capsular polysaccharide (reviewed in Urban et al., 2006).

Neutrophil depletion in mice during protective immune responses does not affect pulmonary fungal burden, indicating that neutrophils are not required for cryptococcal clearance (Wozniak et al., 2012). These data further support the observation by Mednick et al. (2003) that neutropenic mice given a pulmonary *C. neoformans* infection survived significantly longer than control mice with intact neutrophils, therefore indicating that neutrophils are not necessary for protective responses against cryptococcal infection. Furthermore, the presence of neutrophils in the lungs during cryptococcal infection can cause additional damage to the host (Osterholzer et al., 2009a).

In a rat model of *C. gattii* infection, there is an early recruitment of neutrophils into the lungs, but phagocytosis of cryptococci is not observed (Wright et al., 2002). *C. gattii* inhibits or fails to induce migration of neutrophils to the site of infection, thereby impeding an inflammatory response (Cheng et al., 2009). In addition, encapsulated *C. gattii* environmental strains that produced extracellular fibrils (which may be important in cryptococcal cell communication or cryptococcal-host communication) were resistant to neutrophil killing, even when neutrophils produced NETs (Springer et al., 2010).

## Pattern Recognition Receptors

**FIGURE 3:**
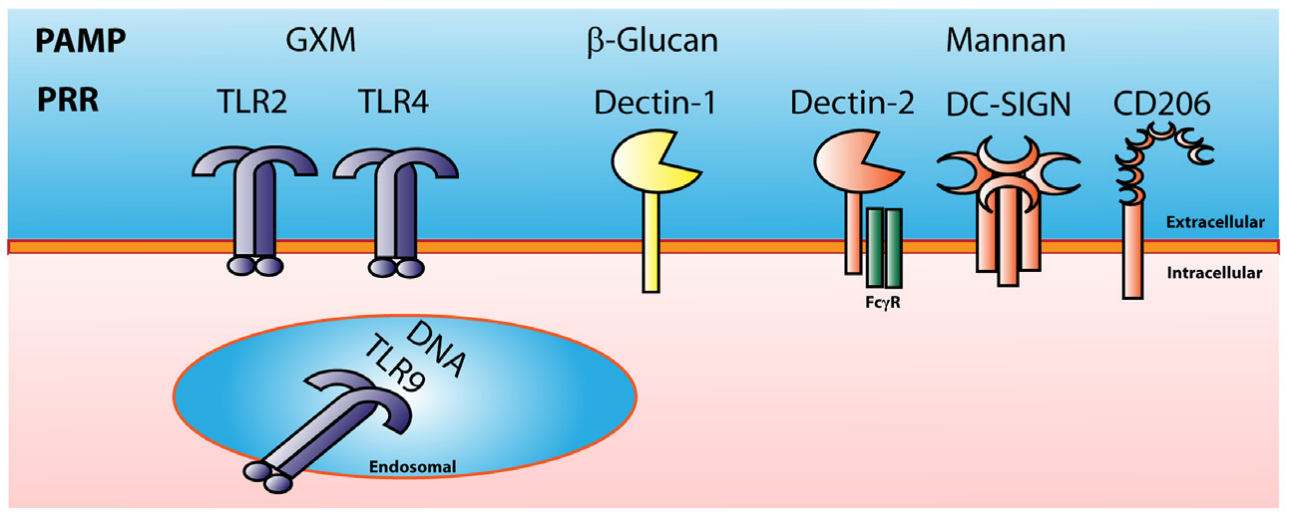
**Cryptococcal Pattern Recognition Receptors.** *Cryptococcus* has multiple pathogen associated molecular patterns (PAMPs) that are recognized by many types of pattern recognition receptors (PRRs). GXM is recognized by TLR2 and TLR4. Cryptococcal mannan can be recognized by the C-type lectin receptors Dectin-2, DC-SIGN, and CD206 or mannose receptor. B-glucans found in the cryptococcal cell wall can be recognized by Dectin-1, and cryptococcal DNA can be recognized by the endosomal receptor TLR9.

In order for the phagocytic cell to take up a pathogen, it has to recognize that the pathogen is foreign. The cell does this by recognition of PAMPs via germline-encoded PRRs present either on the cell surface or within distinct intracellular compartments. These PRRs include TLRs, C-type lectin receptors (CLRs), NOD-like receptors and others. PRRs recognize a wide range of bacterial, fungal, and viral PAMPS, including lipopeptides, peptidoglycan, β-glucans mannan, and pathogen DNA and RNA (**Figure 3**).

One of the main virulence factors of *Cryptococcus* is the anti-phagocytic polysaccharide capsule (Zaragoza et al., 2009). TLR2 and TLR4 have been shown to recognize cryptococcal GXM (Shoham et al., 2001; Yauch et al., 2004, 2005). GXM was shown to bind to both TLR2 and TLR4 with the co-receptor CD14 but failed to activate the MAPK pathway and produce TNF-α (Shoham et al., 2001). TLR2 KO mice were more susceptible to cryptococcal infection than WT control mice; however, there was no difference in survival in C3H/HeJ mice which have a non-functional TLR4, compared to control mice (Yauch et al., 2004). Mice deficient in the adaptor molecule MyD88 (myeloid differentiation primary response gene 88), which is used by all TLRs except TLR3, exhibited a significant increase in mortality compared to WT mice and succumbed to a cryptococcal infection faster than the TLR2 KO mice, suggesting that MyD88 is required for protection against *C. neoformans* (Yauch et al., 2004; Biondo et al., 2005). Peritoneal macrophages from TLR2 KO or MyD88 KO mice exhibit reduced production of TNF-α when cultured with *C. neoformans in vitro* (Biondo et al., 2005). Furthermore, TLR2 KO and MyD88 KO mice showed decreased expression of TNF-α, IFN-γ, and IL-12p40 transcripts in the lungs, brain, and spleen during infection with *C. neoformans* (Biondo et al., 2005). Peripheral blood mononuclear cells (PBMCs) isolated from patients with cryptococcal meningitis exhibited a significant reduction in TLR2 expression compared to healthy control PBMCs and blocking TLR2 on PBMCs led to a reduction in IL-12p70 and IFN-γ when stimulated with *Cryptococcus* (Zhang et al., 2015a). While TLR2 and TLR4 can recognize cryptococcal GXM, these receptors may only play a minor role in protection to *C. neoformans* infection (Nakamura et al., 2006). What is clear is that MyD88 is necessary for protection against *C. neoformans*, suggesting that other TLRs are required.

TLR9 is an endosomal PRR that recognizes unmethylated pathogen CpG DNA following its ingestion and degradation by the immune cell. Uptake of *C. neoformans* leads to the recruitment of TLR9 to the fungal phagosome (Kasperkovitz et al., 2011), and TLR9 KO mice were more susceptible to cryptococcal infection than WT control mice (Wang et al., 2011). Mice treated with the TLR9 antagonist CpG-ODN 3 days before infection with *C. neoformans* strain 52D, a moderately virulent clinical isolate, had reduced fungal burden and pulmonary eosinophilia as well as increased IFN-γ production by CD8^+^ T cells (Edwards et al., 2005). Cryptococcal DNA can activate DCs via TLR9 recognition to produce IL-12p40 and express CD40 (Nakamura et al., 2008). This activity was dependent on the methylation of the DNA as methylase treatment of the DNA led to a reduction of IL-12p40 produced by the DCs. Genetic ablation of TLR9 or MyD88 completely abrogated the effect of cryptococcal DNA on the DCs, demonstrating that DNA recognition and activation by DCs was dependent on TLR9 and MyD88 (Nakamura et al., 2008). Culture supernatants from *C. neoformans* are able to dampen the DC response to cryptococcal DNA (Yamamoto et al., 2011). The inhibitory effects of the supernatants were reduced by heat or trypsin treatment indicating that *C. neoformans* secretes proteinaceous molecules that suppress activation of DCs by cryptococcal DNA (Yamamoto et al., 2011). It was shown that the nucleic acid sequence for cryptococcal URA5 specifically activates DCs through a TLR9-mediated signaling pathway using a mechanism that is different than the canonical CpG motif that is associated with TLR9 signaling (Tanaka et al., 2012). Cryptococcal infection in TLR9 KO mice leads to decreased IFN-γ and TNF-α and an increase in IL-4 compared with WT mice (Zhang et al., 2010). The increased IL-4 in *C. neoformans*-infected TLR9 KO mice led to increased M2 macrophage activation markers arginase and FIZZ1 and a decrease in M1 macrophage marker iNOS compared to WT mice (Zhang et al., 2010). Ablation of TLR9 led to reduction of CD11b^+^ DCs and CCL7 in the lungs during the afferent phase (week 1) and reduced the pulmonary accumulation of CD11b^+^ DCs during the efferent phase [week 3; (Qiu et al., 2012)].

The fungal cell surface is covered in carbohydrates including β-glucans and mannan which are recognized by C-type lectin receptors (CLRs). The β-glucan CLR Dectin-1 has been shown to be important in protection to *Aspergillus*, *Candida*, and *Pneumocystis* infections (Steele et al., 2005; Saijo et al., 2007; Taylor et al., 2007). Dectin-1 has been shown to bind to the β-glucans found on cryptococcal spores (Giles et al., 2009), however, there was no significant difference in disease progression in Dectin-1 KO mice compared to WT mice during cryptococcal infection indicating that Dectin-1 may not be required for host defense to *C. neoformans* (Nakamura et al., 2007).

There are multiple receptors that recognize mannan including the CLRs Dectin-2, MR (CD206), and DC-SIGN. Dectin-2 has been shown to recognize mannan from multiple fungal organisms (Hardison and Brown, 2012). Dectin-2 KO BMDCs incubated with *C. neoformans* failed to produce IL-12p40 and TNF-α as well as failed to increase surface expression of CD86 and MHC II compared to WT BMDCs (Nakamura et al., 2015). In addition, Dectin-2 KO mice infected with *C. neoformans* exhibited higher levels of Th2-type cytokines which are associated with non-protective immune responses compared to infected WT mice (Nakamura et al., 2015). Both MR and DC-SIGN have been shown to recognize heavily mannosylated cryptococcal mannoproteins (Mansour et al., 2006). Human and murine DCs are able to recognize and capture cryptococcal MPs by a mannose receptor (CD206) mediated process (Mansour et al., 2006). MR KO mice succumb to *C. neoformans* infection significantly faster compared to WT mice (Dan et al., 2008a). Cryptococcal MPs in combination with TLR ligands enhanced production of proinflammatory cytokines and chemokines from DCs as well as enhanced MP-specific MHC II-restricted CD4^+^ T-cell responses (Dan et al., 2008a). Increased surface expression of CD206, which is upregulated on M2 macrophages, results in increased phagocytosis but is accompanied by decreased intracellular killing and TNF-α production (Dan et al., 2008a).

## Targeting Macrophages and DCs for Vaccines/Immunotherapies

As demonstrated in the above discussion, innate phagocytes play a pivotal role in the recognition of *Cryptococcus*, anti-fungal activity, and prevention of cryptococcosis. Identification of the specific PRRs utilized by innate immune cells to recognize *Cryptococcus* and lead to the yeast’s internalization and destruction could provide a novel target for treatment. Studies designed to elucidate the host PRR must keep in mind that different immune cells could utilize different PRRs to recognize *Cryptococcus*. In addition, the PAMPs for *C. neoformans* and *C. gattii* may not be the same. More study is required to identify the specific PAMP and PRR combination that elicits protective immune responses.

As *C. gattii* tends to cause disease in immunocompetent individuals, traditional methods of vaccination that elicit a memory T cell response could prove fruitful, however, this has not yet been achieved. High-risk groups for cryptococcosis caused by *C. neoformans*, including AIDS patients, are deficient in CD4^+^ T cells. Nonetheless, recent evidence does suggest that development of a vaccine against *Cryptococcus* is feasible (reviewed in Leopold Wager and Wormley, 2015). It has been shown that CD8^+^ T cells can compensate for the loss of CD4^+^ T cells (Lindell et al., 2005), thus targeting this adaptive immune cell population is possible. Interestingly, immunization of mice with the IFN-γ producing *C. neoformans* strain H99γ leads to protection in B cell deficient mice (Wozniak et al., 2009) as well as WT BALB/c mice that are depleted of both CD4^+^/CD8^+^ T cells during the challenge phase (Wozniak et al., 2011b), suggesting that protection can be achieved in the absence of traditional adaptive immunity. A relatively new concept allows for the generation of “innate memory” or “trained immunity.” This memory occurs in innate cells, including NK cells and monocytes/macrophages in response to *Candida albicans* or simply by “training” via exposure to β-glucans (Quintin et al., 2012). The “trained” monocytes/macrophages have enhanced cytokine recall responses when challenged with *C. albicans*. The protective responses in these cells are non-specific and confer heightened responses following secondary exposure to an antigen, including *C. albicans*. Altogether, this evidence is proof-of-concept that protection can be achieved in immunocompromised individuals.

A novel delivery platform utilizing glucan particles is a promising approach to vaccine design that targets innate phagocytes, including macrophages and DCs. The glucan particles are isolated from baker’s yeast (*Saccharomyces*) and are purified, hollow, and porous cell wall shells composed mostly of β-1,3-glucan (reviewed in Levitz et al., 2015). These stimulate dectin-1 and other PRRs on phagocytes and induce the production of protective cytokines such as IFN-γ and IL-17A (Huang et al., 2009, 2010, 2012, 2013; reviewed in Levitz et al., 2015). The glucan particles can be loaded with proteins, siRNA, DNA, and other small molecules. Immunization with these particles loaded with synthetic peptides of *Coccidioides* results in elevated Th1 and Th17 responses following challenge with *Coccidioides posadasii* (Hurtgen et al., 2012). A recently published study loaded the glucan particles with soluble alkaline extracts from two different attenuated *Cryptococcus* strains, acapsular strain cap59 and strain cda123 which lacks cell wall chitosan. Immunization of mice with the cryptococcal-loaded glucan particles resulted in increased survival following challenge with *C. neoformans* Kn99 and was associated with Th1 and Th17 immune responses (Specht et al., 2015). In addition, mice immunized with glucan particles loaded with cap59 extracts were partially protected against challenge with *C. gattii* strain R265 (Specht et al., 2015). Thus, these studies have identified novel vaccine candidates in the alkaline extracts from *C. neoformans*, and suggest that administration via this delivery system is promising for novel vaccine development.

Immunodominant cryptococcal proteins have recently been identified from both *C. neoformans* and *C. gattii* (Chaturvedi et al., 2013, 2014). Intranasal immunization of mice with cryptococcal cell wall and/or cytoplasmic protein fractions from *C. gattii* or immunodominant protein fractions from *C. neoformans* extended survival following challenge of the respective *Cryptococcus* sp (Chaturvedi et al., 2013, 2014). In addition, certain mannoproteins have been shown to elicit vaccine-mediate immunity, extending survival of *C. neoformans* infected mice (reviewed in Chaturvedi and Wormley, 2013; Levitz et al., 2015). PBMCs from patients who have recovered from cryptococcosis are able to proliferate and produce pro-inflammatory cytokines when stimulated with mannoproteins (Levitz and North, 1997). These proteins and other highly conserved antigens, including heat shock proteins, β-glucan, and glycolytic enzymes, are potential targets for the development of subunit vaccines. Vaccines that utilize recombinant proteins must take into account the posttranslational modifications of these cryptococcal proteins and how that affects immunogenicity.

Specific targeting of macrophage phenotype also has the potential for development of vaccines that would target the host and not the organism, thus limiting selective pressure on the fungus. Treatment of macrophages with IFN-γ results in the M1, fungicidal phenotype, while stimulation with IL-4/IL-13 results in the M2, cryptococcal growth-permissive phenotype (Olszewski et al., 2010; Leopold Wager and Wormley, 2014). It was recently demonstrated that macrophages first polarized to an M2 phenotype with IL-4 can be repolarized to an M1 phenotype following IFN-γ treatment (Davis et al., 2013), demonstrating the plasticity of macrophages in response to their cytokine milieu. These repolarized macrophages are fully functional M1 macrophages, produce NO and are anti-cryptococcal (Davis et al., 2013). Additionally, immunization of mice with the *C. neoformans* IFN-γ-producing strain (H99γ) and challenge with WT *C. neoformans* H99 results in complete protection and M1 macrophage activation (Hardison et al., 2012). This macrophage polarization is occurring even without exogenous IFN-γ produced by the organism, providing evidence for memory T cell responses and possibly for “innate memory” in the macrophages. Recent studies have also shown that DCs are capable of polarizing to a pro-inflammatory DC1 phenotype and anti-inflammatory DC2 phenotype, which may also play a role in cryptococcal immune responses (Guiducci et al., 2005; Cook et al., 2012). Taken together, these studies suggest that targeting the polarization of macrophages and/or DCs toward an anti-cryptococcal phenotype could provide a novel mechanism for the induction of protective responses against *Cryptococcus*. Thus, these innate phagocytes that are critical for the development of protective responses against cryptococci could also be the key to development of novel vaccines and/or immunotherapies to prevent cryptococcosis.

## Conclusion

The interactions between the host and the pathogen are critical for early control of the infection and, thus, the ability of the host to clear the infection. *Cryptococcus* has developed numerous effective strategies to either evade the immune system or to modulate the host cells allowing survival and replication within the phagocyte. There is still much to learn about how the host cells react to *Cryptococcus* and how they allow themselves to be manipulated by the fungus, as well as how the yeast expertly modulates and evades the immune system. In addition, there is an added complexity when the site of infection and the stage of disease is taken into account. Future research should aim to determine the roles of specific molecular interactions between *Cryptococcus* and phagocytes, and how these interactions either contribute to or prevent pathogenesis. A comprehensive understanding of these small, yet substantial interactions will undoubtedly result in effective anti-cryptococcal therapies that will likely be translatable to other intracellular infections which rely so desperately on the early responses of phagocytes.

## Author Contributions

CW, CH, KW, and FW contributed to the writing, editing, and revision of the manuscript.

## Conflict of Interest Statement

The authors declare that the research was conducted in the absence of any commercial or financial relationships that could be construed as a potential conflict of interest.
